# Pea Breeding for Intercropping With Cereals: Variation for Competitive Ability and Associated Traits, and Assessment of Phenotypic and Genomic Selection Strategies

**DOI:** 10.3389/fpls.2021.731949

**Published:** 2021-09-23

**Authors:** Paolo Annicchiarico, Nelson Nazzicari, Tommaso Notario, Cristina Monterrubio Martin, Massimo Romani, Barbara Ferrari, Luciano Pecetti

**Affiliations:** Council for Agricultural Research and Economics (CREA), Research Center for Animal Production and Aquaculture, Lodi, Italy

**Keywords:** genomics, GWAS, morphophysiological traits, phenology, *Pisum sativum*, plant competition dynamics, selection efficiency, selection index

## Abstract

Mixed stand (MS) cropping of pea with small-grain cereals can produce more productive and environment-friendly grain crops relative to pure stand (PS) crops but may require selection to alleviate the pea competitive disadvantage. This study aimed to assess the pea variation for competitive ability and its associated traits and the efficiency of four phenotypic or genomic selection strategies. A set of 138 semi-leafless, semi-dwarf pea lines belonging to six recombinant inbred line populations and six parent lines were genotyped using genotyping-by-sequencing and grown in PS and in MS simultaneously with one barley and one bread wheat cultivar in two autumn-sown trials in Northern Italy. Cereal companions were selected in a preliminary study that highlighted the paucity of cultivars with sufficient earliness for association. Pea was severely outcompeted in both years albeit with variation for pea proportion ranging from nearly complete suppression (<3%) to values approaching a balanced mixture. Greater pea proportion in MS was associated with greater total yield of the mixture (*r* ≥ 0.46). The genetic correlation for pea yield across MS and PS conditions slightly exceeded 0.40 in both years. Later onset of flowering and taller plant height at flowering onset displayed a definite correlation with pea yield in MS (*r* ≥ 0.46) but not in PS, whereas tolerance to ascochyta blight exhibited the opposite pattern. Comparisons of phenotypic selection strategies within or across populations based on predicted or actual yield gains for independent years indicated an efficiency of 52–64% for indirect selection based on pea yield in PS relative to pea yield selection in MS. The efficiency of an indirect selection index including onset of flowering, plant height, and grain yield in PS was comparable to that of pea yield selection in MS. A genome-wide association study based on 5,909 SNP markers revealed the substantial diversity of genomic areas associated with pea yield in MS and PS. Genomic selection for pea yield in MS displayed an efficiency close to that of phenotypic selection for pea yield in MS, and nearly two-fold greater efficiency when also taking into account its shorter selection cycle and smaller evaluation cost.

## Introduction

Intercropping, i.e., the simultaneous cultivation of two or more crop species in the same field, provides agronomic benefits that have long since been noticed (e.g., Darwin, 1859). This technique has largely been adopted in traditional subsistence agriculture (Vandermeer, 1989; Altieri, 2004), while remaining widespread in modern agriculture only for some perennial forages, e.g., white clover-grass mixtures (Haynes, 1980). However, the association of annual legumes with cereals may become a cornerstone of the necessary agroecological transition of modern agriculture, to exploit plant functional diversity for a sustainable intensification aimed to raise crop yields, yield stability, and/or crop quality while simultaneously enhancing ecosystem services and reducing adverse environmental impacts. Indeed, meta-analysis studies encompassing different interspecific mixtures and cropping regions indicated that intercropping, compared with the mean value of the sole crops of its component species, displayed an average yield advantage of 22–30% (Yu et al., 2015; Himmelstein et al., 2017; Martin-Guay et al., 2018) along with distinctly greater crop yield stability (Raseduzzaman and Jensen, 2017). The main reason for these advantages lies in more efficient utilization of light, water, or nutrients by a complementary plant foraging pattern that implies lower interspecific competition than intraspecific competition (Lithourgidis et al., 2011; Brooker et al., 2015). The intercropping of grain legumes with cereals, which accounts for the large majority of the scientific reports for annual crops (Yu et al., 2015; Raseduzzaman and Jensen, 2017), can exploit species complementarity effects for nitrogen use (atmospheric N_2_ for legumes and soil N for cereals) (Schmidtke et al., 2004; Bedoussac et al., 2015; Rodriguez et al., 2020) that allow to reduce crop N fertilization and, thereby, greenhouse gas emissions, energy consumption, and N leaching into fresh water (Jensen et al., 2020). While increasing and stabilizing crop yields in both high- and low-input systems (Li et al., 2020), these mixtures under low soil N availability (as it may be in organic systems) also lead to greater cereal protein content (Gooding et al., 2007; Bedoussac and Justes, 2010). Additional advantages of grain legume-cereal intercrops relative to sole crops may include the reduction of pests and diseases caused by dilution of the host density (Boudreau, 2013), better control of weeds (Liebman and Dyck, 1993; Corre-Hellou et al., 2011), and the ability of some species to chemically mobilize and make available for the companion species one or more limiting soil nutrients such as phosphorus, iron, zinc, or manganese (Zhang and Li, 2003; Li et al., 2014). The increasing awareness of all these advantages is leading to increasing intercropping of grain legume-cool season cereals in Europe, particularly in organic systems (Schneider et al., 2015).

While offering several opportunities, grain legume-cereal intercropping also poses various technical challenges that hinder its adoption by farmers, among which the development of suitable cultivars and better mechanical implementation stand out for importance (Martin-Guay et al., 2018). A balanced competition between component species is required for the display of agroecological benefits (Corre-Hellou et al., 2006) and, when relevant, the achievement of certain crop quality characteristics (e.g., protein content). However, asymmetrical competition leading to a competitive advantage of cereals has frequently been reported as a consequence of weaker competitive ability by legumes. This emerged for pea in different mixtures and cropping regions (Jensen, 1996; Corre-Hellou et al., 2006; Lithourgidis et al., 2011; Annicchiarico et al., 2017), with the exception of one experiment whose management (adoption of a relatively weak competitor such as barley associated with lack of N fertilization) limited the cereal aggressiveness (Hauggaard-Nielsen and Jensen, 2001). Competitive disadvantage was also reported for other cool-season annual legumes associated with small-grain cereals, such as lentil (Schmidtke et al., 2004), white lupin (Mariotti et al., 2009) and vetches (Annicchiarico et al., 2017), and warm-season legumes such as common bean, cowpea, soybean, pigeonpea, or groundnut intercropped with maize or sorghum (Ofori and Stern, 1987; Santalla et al., 2001; Boukar et al., 2015).

The size of the genetic correlation between pure stand (PS) and mixed stand (MS) conditions for performance of a reasonably large genotype sample of a focus species describes synthetically the intrinsic consistency of genotype response across growing conditions and contributes crucially to assess the predicted efficiency of different phenotypic selection strategies aimed to improve the species performance in MS (Annicchiarico et al., 2019a). These strategies may encompass direct selection for yield in MS, indirect selection in PS for yield (which implies lower cost than MS because there is no need for separation or proportion assessment of the focus species), and indirect selection in PS based on morphophysiological traits associated with yield and competitive ability in MS (Annicchiarico et al., 2019a). By the third strategy, traits that are not highly correlated to each other and that feature high correlation with performance in MS, low evaluation cost, and moderately high broad-sense heritability and repeatability across locations and/or cropping years are pooled into a selection index applied to material evaluated in PS (Annicchiarico, 2003). Breeding for intercropping was studied on large genotype numbers, and produced documented improvements, only for perennial legumes, especially white clover (e.g., Annicchiarico and Proietti, 2010). In contrast, investigations on grain legumes were usually based on small numbers of cultivars or breeding lines, thereby producing data that may support selection strategies by revealing genotype variation in competitive ability and different top-performing genotypes across PS and MS conditions [as in Baxevanos et al. (2017) for pea in MS with oat] but could hardly be used to compare breeding strategies in terms of selection efficiency. Likewise, traits associated with competitive ability, whose mechanisms may also contribute to complementarity of the associated species (Litrico and Violle, 2015), were poorly investigated in grain legumes (Annicchiarico et al., 2019a).

Breeding for intercropping is challenged by the commercial interest of selecting for a range of possible companion species and varieties in a cost-efficient manner. Results for perennial legumes indicated that general-compatibility effects (which express consistent yield responses across different associations) are definitely larger than specific-compatibility effects (which express association-specific yield responses) (Holland and Brummer, 1999; Maamouri et al., 2017), and that the latter effects are affected by the difference in competitive ability more than by the species of the associated partner (Annicchiarico and Piano, 1994). Because of that, selection in one MS condition in which the associated partner was represented by a few highly competing genotypes of different grass species sown together as a pooled tester in Annicchiarico (2003) proved to be a low-cost means to select white clover for general compatibility, as indicated by improvements of clover yield and competitive ability expressed consistently across a set of different species and varieties (Annicchiarico and Proietti, 2010). A recent study indicated that general-compatibility effects are much larger than specific-compatibility effects also for pea-barley associations (Haug et al., 2020). For breeding and cultivation of annual legumes to be intercropped for grain production, a further challenge is the identification of cereal companions whose maturity date is sufficiently close to that of the legume cultivar to be selected or grown.

Genomic selection was indicated as a priority theme for research aimed to define new breeding strategies for intercropping, because of the costs and complexity of phenotypic selection in MS conditions (Annicchiarico et al., 2019a). Genomic selection, which implies the construction of a statistical model based on phenotyping and genotyping data of a germplasm sample representative of the target genetic base and its subsequent application to predict breeding values of a large set of independent genotyped individuals (Heffner et al., 2009; Lorenz et al., 2011), aims to reduce selection costs by partly substituting for phenotypic selection. Its cost-efficient application to plant breeding has greatly been enhanced by recent sequencing techniques, such as genotyping-by-sequencing (GBS; Elshire et al., 2011), that allow large germplasm sets to be genotyped by thousands of single nucleotide polymorphism (SNP) markers at a relatively low cost. Pioneer studies for pea suggested greater genetic gain per unit time of genomic over phenotypic selection for improving grain yield under PS conditions in moisture-favorable (Annicchiarico et al., 2019b) and severely drought-prone target regions (Annicchiarico et al., 2020). Genomic selection out-performed phenotypic selection in breeding for intercropping in a study based on stochastic simulation data (Bančič et al., 2021), but no experimental assessment of the value of genomic selection for intercropping is available.

This study focused on 144 pea inbred lines, of which 138 were randomly sorted out in equal proportions from six recombinant inbred line (RIL) populations issued by crosses between elite semi-dwarf, semi-leafless cultivars and six were parent lines. This material was genotyped by GBS and was grown in PS and MS in Northern Italy in two cropping years. MS implied the simultaneous association of pea with one barley and one bread wheat cultivar selected by a prior phenology assessment study. The main objectives of this study were (a) to investigate the pattern and extent of pea genetic variation for competitive ability against cereals, (b) to assess the consistency of pea grain yield responses across MS and PS conditions according to estimates of genetic correlation and information on genomic regions associated with yield responses in a genome-wide association study (GWAS), (c) to identify traits associated with pea competitive ability, and (d) to compare four selection strategies for pea performance in intercropping, namely, direct phenotypic selection for grain yield in MS, indirect phenotypic selection based on grain yield in PS, indirect selection based on an index of traits associated with pea competitive ability assessed in PS, and genomic selection for grain yield in MS, in terms of predicted or actual yield gains.

## Materials and Methods

### Definition of Cereal Cultivars With Acceptable Maturity Date for Use as Testers

All experiments were carried out under field conditions in Lodi, Northern Italy (45°19′ N, 9°30′ E, 81 m a.s.l.), which features sub-continental climate and sandy-loam soils with pH around 6.5. Pea intercropping was foreseen with barley or triticale for feed production, and with bread or durum wheat mainly for food production. A preliminary experiment was set up to assess the heading and maturity dates and the plant height at heading of 14 cultivars of bread wheat, three of barley, two of triticale and one of durum wheat, in relation to onset of flowering and maturity dates and plant height at onset of flowering of a reference set of 14 pea genotypes that were concurrently evaluated (Supplementary Table 1). The set of bread wheat genotypes included nine recent commercial varieties grown in Italy, three breeding lines (A208, A210, and F426) chosen among the earliest-maturing ones bred by INRAE's UMR Génétique Quantitative et Évolution of Le Moulon (France), and the historical Italian cultivars San Pastore bred in 1929 (still adopted by Italian organic farmers) and Spada bred in 1985 because of their known outstanding earliness. The set of cereal material was completed by three elite modern varieties of barley, and recent commercial varieties of triticale or durum wheat that were described as very early. The pea genotypes included the commercial varieties Alliance, Attika, Dove, Guifilo, Isard, and Kaspa, which acted as parent lines of the six RIL populations that provided the genetic base for subsequent experiment work, and eight breeding lines that expressed the range of variation for phenology and plant height observed in the prior multi-locational testing by Annicchiarico et al. (2019b) of 306 lines issued by three connected crosses among the varieties Attika, Isard, and Kaspa. All pea genotypes were semi-dwarf, semi-leafless plant types.

The genotypes were evaluated as single rows 2 m long and 0.37 m apart, according to a group balanced block design (Gomez and Gomez, 1984) with three replications, of which cereal and pea material represented the groups. The sowing rate was 260 seeds/m^2^ for bread wheat, 222 seeds/m^2^ for durum wheat and triticale, 186 seeds/m^2^ for barley, and 55 seeds/m^2^ for pea. The experiment was sown at the end of October 2017. The total rainfall during the growing period (November-June) was 622 mm. The number of frost days was 57, with a minimum absolute temperature of −11.6°C.

The experimental data underwent an analysis of variance (ANOVA) holding the fixed factors group and genotype within group and the random factor block aimed to compare cereal vs. pea germplasm groups, and separate ANOVAs aimed to assess the variation within cereal and pea germplasm groups. The results assisted the selection of the cereal cultivars used as testers in the following work.

### Evaluation of Pea Inbred Lines in Pure Stand and Mixed Stand

A set of 144 semi-leafless, semi-dwarf inbred lines was evaluated under PS and MS in Lodi during the cropping seasons 2018–19 and 2019–20. The set included 23 lines randomly sorted from each of six RIL populations, and the six parent lines of the populations. The populations originated from the following crosses: (a) Attika × Isard, (b) Kaspa × Attika, (c) Kaspa × Isard, (d) Dove × Attika, (e) Attika × Guifilo, (f) Alliance × Isard. The parent lines were selected within a large number of international cultivars because of their high and stable grain yield and only moderate phenological differences across the environments of northern and southern Italy (Annicchiarico, 2005; Annicchiarico and Iannucci, 2008). The large use of Attika as a parent in these crosses was due to its high competitive ability against weeds under organic management (Annicchiarico and Filippi, 2007), which may relate to competitive ability under intercropping (Annicchiarico et al., 2019a).

The first cropping season adopted an early sowing (October 25), a cereal tester represented by the mixture of the barley cultivar Atlante with the tall early wheat cultivar San Pastore, and a pre-sowing fertilization of 50 kg/ha of N along with 75 kg/ha P_2_O_5_ and 100 kg/ha K_2_O. In order to widen the environmental variation between test environments, the second cropping year adopted crop establishment conditions that were expected to be more favorable for pea growth in MS relative to the first year, namely, a late sowing (December 10), a cereal tester represented by the mixture of the barley cultivar Atlante with the short early wheat cultivar Spada, and a pre-sowing fertilization including 25 kg/ha of N along with 75 kg/ha P_2_O_5_ and 100 kg/ha K_2_O. Each experiment was laid out as a split-plot with three replications holding growing condition (MS or PS) on main plots and pea lines on subplots. The seed rate of pea in MS was half of that adopted in PS (40 vs. 80 seeds/m^2^). The cereal seed rates in MS were 75 seeds/m^2^ for barley and 100 seeds/m^2^ for wheat, corresponding to 25% of the ordinary rate in the region for each species (which implied a halved seed rate for the whole of the cereal tester in MS relative to the ordinary rate in PS). MS plots were 2.0 m long and 1.36 m wide, and PS plots were 1.0 m long and 1.36 m wide, to keep constant the number of pea test seeds in each condition. All plots included 6 rows, blending pea and cereal seeds on each row in MS as done ordinarily by local farmers for pea-cereal intercrops. Seedbed preparation by plowing and harrowing was the same for MS and PS, whereas chemical weed control [Stomp^®^ 330 E (a.i. Pendimethalin at 307 g/L) at 4.5 L/ha] was applied only to PS to limit the relatively large growth of weeds expected in this condition. The first cropping year, compared with the second year, featured greater winter cold stress (61 vs. 53 frost days; absolute minimum temperature of −12.0 vs. −10.9°C) and more rainfall, especially in late spring (April-May rainfall of 233 mm vs. 66 mm).

The following traits of pea lines were recorded on PS plots: (a) winter plant survival, based on plant counts at the onset and the end of winter; (b) onset of flowering, as number of days from April 1 to when 50% of plants in the plot had at least one fully open flower; (c) mean plant (canopy) height at onset of flowering; (d) susceptibility to the ascochyta blight disease complex (*Didymella* spp.), assessed in spring on a visual 9-level scale ranging from 1 (no damage) to 9 (plant mortality > 20%) (recorded in the first year, the only year that featured a sizeable disease incidence); (e) crop maturity (as the number of days from April 1); (f) plant height at crop maturity, measured on two random outstretched plants; (g) dry grain yield, after combine-harvesting of the plots at crop maturity (PS) and assessment of seed moisture on a random sample of 100 seeds oven-dried at 90°C for 4 days; (h) dry individual seed weight, assessed on the seed sample used for seed moisture determination. The traits recorded on MS plots included the dry grain yield of pea and of the pooled cereal tester, the total (pea + cereal) dry yield of the mixture and the proportion of pea dry yield on total yield, computed after harvesting the plot fresh seed and using a seed sample of 100 g for separation and dry weight assessment of the relative proportion of pea and cereal components. Onset of flowering, mean plant height at onset of flowering, and dry individual seed weight were recorded on MS plots only in the second year, to assess their consistency across MS and PS conditions in one test year. The ratio between pea yield in MS and pea yield in PS, defined for MS plots by imputing the mean yield in PS of each line, provided an additional variable aimed to highlight genotype × growing condition interaction responses leading to relatively better response in MS. Pea yield in MS was doubled prior to ratio computation, in order to express the ratio with respect to the same growing area (thus, assuming a halved area for pea in MS relative to pea in PS).

### Statistical Analysis of Phenotypic Data of Pea Inbred Lines in Pure Stand and Mixed Stand

A preliminary analysis of variance (ANOVA) including the factors pea line and block was performed on data of separate growing conditions (PS or MS) and cropping years to verify the occurrence of genetic variation among lines for each trait. A second ANOVA including the factors pea line, growing condition, and block was performed on the data of separate cropping years according to the split-plot lay-out to verify the variation between conditions and the occurrence of genotype × condition interaction for traits recorded in both conditions. A third ANOVA including the factors pea line, cropping year, and block within year was performed separately for data recorded in PS or MS in both years to verify the variation between years and the occurrence of genotype × year interaction. This ANOVA was also applied to a composite trait represented by a selection index including traits observed in PS. One last ANOVA including the factors pea line, growing condition, cropping year, and block within year was performed on pea grain yield data mainly to verify first- and second-order interactions of the genotype factor (while testing the variation for condition and condition × year interaction using condition × block within year as the error term). Variance components were estimated by a Restricted Maximum Likelihood (REML) method for the same ANOVA with respect to genotype (considered as a random factor) and its interactions with growing condition and year.

Relationships between traits were investigated by simple correlation analysis of genotype values. Statistical differences between correlation coefficients between PS and MS conditions were assessed by the *u* test described by Dagnelie (1975).

An index of indirect selection for pea yield in MS was defined from pea characters observed in PS, using line values previously averaged across cropping years. The weights of the variables in the index were estimated from partial regression coefficients as reported in Wricke and Weber (1986). The choice of the best index was based on the significance of partial regression coefficients within a stepwise multiple regression approach, allowing for the inclusion of an additional trait in the index when the trait featured *P* < 0.05 significance and increased the regression *R*^2^ by at least 0.02.

Three pea selection strategies for pea yield in MS, namely, direct selection in MS, indirect selection in PS based on pea yield, and indirect selection in PS based on the selection index, were first compared according to predicted yield gains estimated separately from the data of each cropping year. Recalling that the genetic parameters for a selection index can be estimated in the same manner as those for individual traits (Lin, 1978), the relative efficiency *E*_*R*_ of indirect selection in PS vs. direct selection in MS, expressed in percentage, was estimated by the following equation (Falconer, 1989):


$$\begin{array}{l} E_{R}=\left[\left(H_{PS}/H_{MS}\right)r_{g}\right]\times 100 \end{array}$$

where *H*_*PS*_ and *H*_*MS*_ are the square root of the broad-sense heritability on a line mean basis (*H*^2^) for the relevant selection criteria in PS and MS, respectively, and *r*_*g*_ is the genetic correlation between the two criteria. Heritability values were computed from genotypic ($s_{g}^{2}$) and experiment error ($s_{e}^{2}$) components of variance estimated by a REML method and *n* number of line replicates per condition by the equation: *H*^2^ = $s_{g}^{2}$ / ($s_{g}^{2}$ + $s_{e}^{2}$ / *n*). An approximate standard error was computed as reported in Uddin et al. (1994). The genetic correlation was estimated as described by Robertson (1959) for traits assessed in different experiment units. The consistency of pea line response across conditions as described by the genetic correlation was also estimated for the three morphophysiological traits of pea recorded in both conditions in the second cropping year.

The described comparison of selection criteria was limited to single-year results without taking into account the possible differences among criteria for the extent of genotype × year (GY) interaction. We verified the significance of this interaction for each selection criterion by ANOVA and assessed the extent of the interaction by the genetic correlation for pea line response across cropping years according to Itoh and Yamada (1990) for one trait assessed in different environments. Broad-sense heritability values taking also account of GY interaction were computed from genotypic ($s_{g}^{2}$), GY interaction ($s_{gy}^{2}$), and experiment error ($s_{e}^{2}$) components of variance estimated by a REML method, *y* cropping years, and *n* number of line replicates per condition by the equation:


$$\begin{array}{l} H^{2}=s_{g}^{2}/\left(s_{g}^{2}+s_{gy}^{2}/y+s_{e}^{2}/y\;n\right). \end{array}$$

A comparison of selection strategies based on predicted genetic gains that accounted for GY interaction effects was based on the view of each selection criterion in a given year (including that based on yield in MS) as an indirect selection criterion for the target trait represented by pea yield in MS in the other year. In this context, the size of the phenotypic correlation between pea line values for a given criterion in one year and pea line yields in MS in the other year is proportional to the expected genetic gain for the target trait provided by the relevant criterion (Cooper et al., 1996). We estimated phenotypic correlations using by turns one cropping year as the selection environment and the other year as the target environment, and expressed the relative efficiency *E*_*R*_ of selection in PS vs. selection in MS as a function of the average correlation across years for selection in PS based on the relevant criterion (*r*_*PS*_) and selection based on pea yield in MS (*r*_*MS*_) by the following equation:


$$\begin{array}{l} E_{R}=\left(r_{PS}/r_{MS}\right)\times 100. \end{array}$$

One last comparison of phenotypic selection strategies was based on actual yield gains when adopting one year for selection of two lines out of 23 for each of the 6 RIL populations and the other year for estimation of yield gains obtained by the selected material over the mean value of the six parent lines of the RIL populations, using by turns one year for selection and the other year for yield gain assessment. The relative efficiency *E*_*R*_ of selection in PS vs. selection in MS was estimated by the following equation:


$$\begin{array}{l} E_{R}=\left(G_{SC}/G_{MS}\right)\times \;100 \end{array}$$

where *G*_*SC*_ and *G*_*MS*_ are yield gains for the relevant criterion for PS selection and the selection based on yield in MS, respectively.

All analyses of phenotypic data were carried out using SAS/STAT® software (SAS Institute, 2011).

### DNA Isolation, GBS Library Construction, Sequencing, and SNP Calling

Pea leaf green tissues for DNA extraction were collected, flash frozen in liquid nitrogen, and stored at −80°C before analyses. Genomic DNA was extracted from 6 bulked plants per genotype using the DNeasy Plant Mini Kit (Qiagen) and checked for integrity on 1% agarose gel. DNA quantitation was performed by means of the Quant-iT^TM^ PicoGreen dsDNA assay kit (Life Technologies, P7589). The GBS data were generated by the Elshire Group Ltd. according to the protocol described by Elshire et al. (2011) with the following modifications: 100 ng of genomic DNA were used, 3.6 ng of total adapters were used, the genomic DNAs were restricted with *ApeK*I enzyme, and the library was amplified with 14 PCR cycles. Library sequencing was performed using the Illumina HiSeq X platform and paired-end runs (2 × 150 bp).

The SNP calling was performed using the dDocent pipeline (Puritz et al., 2014), aligning reads on the pea reference genome (Kreplak et al., 2019) release v1a as downloaded from https://urgi.versailles.inra.fr/download/pea/. The resulting vcf file was filtered for quality using *vcftools* (Danecek et al., 2011) with options—remove-indels—minQ 30 —non-ref-af 0.001—max-non-ref-af 0.9999—max-missing 0.3. The resulting filtered file was transformed in a 012 SNP matrix and further filtered for minor allele frequency (MAF) >5% and several levels of maximum missing rate per marker (1, 3, 5, 10%) and per genotype (10, 25, 50%). Markers with heterozygosity ratio >95% were discarded as well. Missing data points in the resulting SNP matrices were imputed according to the k-nearest neighbors imputation (KNNI) method (Nazzicari et al., 2016).

### Genome-Enabled Predictions and Comparison of Genomic vs. Phenotypic Selection Strategies

Genomic selection models were constructed from phenotypic data represented by best linear unbiased prediction (BLUP) values of pea grain yield in MS calculated as described in DeLacy et al. (1996). We considered various genomic regression models either capable of accepting SNP matrices as input, such as Ridge regression BLUP, Bayes A, Bayes Cπ and Bayesian Lasso, or requiring a kinship matrix, such as Genomic best linear unbiased prediction (G-BLUP) and Reproducing Kernel Hilbert Space (Lorenz et al., 2011; Wang et al., 2018). The kinship matrix was computed according to Astle and Balding (2009). No extra covariates were used. All regression models were implemented using the GROAN R package (Nazzicari and Biscarini, 2018).

Predictive ability was assessed as Pearson's correlation between observed and genomically predicted phenotypes according to inter-environment predictions based on model training in one test year and model validation in the other year. Inter-environment predictions were relative to three scenarios, namely, intra-RIL population predictions, inter-RIL population predictions, and predictions relative to the entire set of material (i.e., without distinction among populations). Intra-population inter-environment predictions were also used for a two-stage process of model tuning, in which the first stage aimed to select the thresholds of missing rate per marker and per genotype according to predictive ability values issued by Ridge regression BLUP, and the second aimed to select the statistical model on the ground of model predictive abilities for the selected configuration of marker and genotype missing rates. Intra-population predictions, and predictions for the whole genetic base, adopted a five-fold stratified cross validation scheme with modifications. In particular, model training was based on yield data of a random set of nearly 80% of the lines belonging to each of the six RIL populations (namely, 18 lines out of 23), using yield data in the other year of the remaining 20% of lines (5 lines) of each population for predictive ability assessment. The six parent lines were always added to the training set. This cross validation process was repeated 100 times by ensuring that each line from each population was included in the validation set a constant number of times, averaging the results across repetitions and repeating the whole analysis for each training year. This analysis was also used to assess actual yield gains derived from genomic selection by selecting two top-yielding lines per population according to genome-enabled breeding values averaged across repetitions and assessing the gains as yield difference in the other test year of the selected material relative to the mean value of six parent lines, using by turns one year for selection and the other for yield gain assessment. The relative efficiency *E*_*R*_ of genomic selection was estimated from gains for the relevant genomic selection criterion *G*_*SC*_ and for phenotypic selection for yield in MS (*G*_*MS*_) according to the following formula:


$$\begin{array}{l} E_{R}=\left(G_{SC}/G_{MS}\right)\times 100. \end{array}$$

Inter-population inter-environment predictions assumed model training based on data in one year of all lines of five non-target RIL populations and the set of parent lines, and model validation based on data in the other year of all lines of the target population. This assessment (which implied no need for cross validation) was repeated for each possible target population and training year.

### Genome-Wide Association Study

A GWAS was carried out for pea yield in MS and in PS using line values averaged across the two cropping years. We used the same levels of filtering for the genotype matrix that optimized genome-enabled predictions. The association study was implemented using the statgenGWAS R package (van Rossum and Kruijer, 2020), including genomic control and the RIL population incidence matrix as a covariate. Significance level thresholds for multiple testing were established *via* Bonferroni method. Non-aligning markers were placed on a fictitious chromosome 99 for display purposes.

## Results

### Definition of Cereal Cultivars With Acceptable Maturity Date for Use as Testers

On average, cereal material headed about 4 days earlier than pea mean onset of flowering, and exhibited nearly 14 dd later maturity and 17 cm taller plant stature than pea germplasm (*P* < 0.01; Supplementary Table 1). Barley tended to be earlier-maturing than the other cereal species, but all cereal genotypes displayed at least 4-day later maturity than the mean maturity of pea material (Supplementary Table 1). The earliest genotypes, namely the barley cultivar Atlante and the bread wheat cultivars Spada and San Pastore, were selected as testers, because their maturity time (albeit suboptimal) did not exceed one week relative to the pea mean maturity. Atlante featured fairly high plant stature (92 cm), Spada short stature (69 cm), and San Pastore tall stature (102 cm). As anticipated, the seed mixture of Atlante and San Pastore acted as cereal tester in the first cropping year, and that of Atlante and Spada (expected to exert somewhat lower competitive ability on pea) acted as cereal tester in the second year. Pea cultivar and breeding line groups displayed similar phenology, along with fairly large within-group variation for onset of flowering and plant height and modest variation for maturity date (Supplementary Table 1).

### Phenotypic Variation in Mixed Stand and Pure Stand and Comparison of Phenotypic Selection Strategies

The first cropping year, featuring earlier sowing and wetter spring, had over 30% greater mean yield of pea in PS and mean total (pea + cereal) yield in MS relative to the second year (Table 1). This result was associated with a prolonged reproductive stage of the crops favored by moisture-favorable conditions, as indicated by pea in PS showing slightly later mean crop maturity along with much earlier mean onset of flowering in the first year compared with the second year (Table 1). On average, the total yield of the mixed crop was about 4% higher in the first year and 2% higher in the second year relative to pea yield in PS (Table 1). On average, pea was at severe competitive disadvantage with associated cereals in both years, although the disadvantage was greater in the first year than in the second one (0.152 vs. 0.214 mean pea proportion on total grain yield) as expected from its less favorable conditions for pea growth in MS (as determined by earlier sowing, taller wheat companion, and higher N fertilization). Severe mean depression of pea yield in MS relative to PS was highlighted by the MS to PS ratio of pea yield per unit area, which fell below 0.5 in both years (Table 1) (while equalling unity in the case of no yield depression).

**Table 1:** Mean and range values of pea traits in pure stand (PS) and pea and associated cereal traits in mixed stand (MS) for 144 pea inbred lines grown in two cropping years.

		**2018–19^c^**				**2019–20^c^**			
**Trait^a^**	**Condition^b^**	**Mean^d^**		**Min**.	**Max**.	**Mean^d^**		**Min**.	**Max**.
Pea grain yield (t/ha)	PS	6.223	a A	1.571	9.286	4.686	b A	2.282	6.904
Pea grain yield (t/ha)	MS	1.000	a B	0.174	2.126	1.071	a B	0.092	2.688
Associated cereal grain yield (t/ha)	MS	5.494	a	4.103	6.584	3.719	b	2.436	5.523
Total (pea + cereal) yield (t/ha)	MS	6.494	a	5.378	7.580	4.790	b	3.158	6.970
Pea proportion	MS	0.152	a	0.029	0.297	0.214	a	0.029	0.458
Pea MS/PS grain yield ratio	MS	0.345	a	0.058	0.902	0.478	a	0.042	1.224
Pea onset of flowering (dd from Apr 1)	PS	12.7	b	1.0	24.7	27.0	a A	23.0	31.0
Pea onset of flowering (dd from Apr 1)	MS	–		–	–	27.8	A	24.3	31.0
Pea plant height at onset of flowering (cm)	PS	62.0	a	27.7	99.0	46.1	b A	32.6	60.0
Pea plant height at onset of flowering (cm)	MS	–		–	–	50.1	A	33.3	68.3
Pea individual seed weight (g)	PS	0.146	b	0.101	0.213	0.198	a A	0.148	0.292
Pea individual seed weight (g)	MS	–		–	–	0.194	A	0.137	0.276
Pea maturity date (dd from Apr 1)	PS	64.5	a	59.0	68.5	61.9	b	59.0	65.1
Pea plant height at maturity (cm)	PS	120.4	a	77.5	162.3	54.8	b	34.3	79.6
Pea winter plant survival (proportion)	PS	0.978		0.849	1.000	–		–	–
Pea susceptibility to ascochyta blight (scale 1–9)	PS	4.1		3.0	5.3	–		–	–

a *Line variation within experiment and growing condition always significant at P < 0.01, except for associated cereal yield in 2019–20 significant at P < 0.05*.

b * Cereal tester in MS formed by mixing one barley cultivar and one bread wheat cultivar*.

c * Sowing time, cereal companions and pre-sowing N fertilization expected to be more favorable for pea in MS in 2019–20 relative to 2018–19*.

d * Means followed by different lower-case letter differ between cropping years in the same growing condition at P < 0.05; means followed by different capital letter differ between growing conditions in the same cropping year at P < 0.05*.

Pea line variation within the cropping year and growing condition was significant at *P* < 0.01 for all traits except associated cereal yield in MS in 2019–20, which achieved *P* < 0.05 significance, and pea winter plant survival and susceptibility to ascochyta blight in the second year, in which the absence of pea line variation was associated with climatic conditions that did not favor the occurrence of winter plant mortality and foliar diseases. The range of genotype variation for winter mortality in the first year was modest albeit significant (Table 1). In contrast, large variation was observed in both years for most traits recorded in PS or MS, including pea and total yield in MS, pea competitive ability as expressed by pea proportion in MS, and the pea yield ratio between MS and PS (Table 1). The variation for pea proportion ranged from pea lines that were nearly suppressed (values <3%) to lines competitive enough to approach a balanced mixture (values close to 30% in the first year and 45% in the second year: Table 1). The poorest-competing pea material exhibited about twenty-fold reduction of grain yield per unit area in MS relative to PS (as indicated by ratio values close to 0.05), whereas the best-competing material suffered a modest or nil yield reduction in MS (ratio values close to unity) (Table 1).

The variation for pea proportion in MS was nearly coincident with that for pea yield in MS, based on the correlation close to unity of these traits (Table 2). This finding reinforced the choice of pea yield in MS as the focus trait for pea selection targeted to intercropping. The correlation of the pea MS/PS grain yield ratio with pea yield and pea proportion in MS was high although not close to unity (Table 2), as the ratio expressed genotype × growing condition interaction effects while the other two traits expressed performance in MS as derived from the combination of positive genotype × growing condition interaction effects and intrinsic yielding ability as displayed in PS. Importantly, greater pea proportion in MS was correlated with greater total yield of the mixture (*r* ≥ 0.46; Table 2), revealing that greater pea yield and competitive ability in MS tends to produce mixtures that are not only more balanced but also more productive (albeit in the presence of some trade-off between pea and cereal yields highlighted by a low inverse correlation between these traits: Table 2).

**Table 2:** Phenotypic correlation of pea grain yield or pea proportion in mixed stand with cereals (MS) with pea or cereal yield traits in MS or pea yield in pure stand (PS), for 144 pea inbred lines grown in two cropping years.

	**Pea grain yield in MS**		**Pea proportion in MS**	
**Trait**	**2018–19**	**2019–20**	**2018–19**	**2019–20**
Pea proportion in MS	0.98^**^	0.96^**^	–	–
Associated cereal grain yield in MS	−0.31^**^	0.04 NS	−0.46^**^	−0.16^*^
Total (pea + cereal) grain yield in MS	0.60^**^	0.77^**^	0.46^**^	0.62^**^
Pea MS/PS grain yield ratio	0.64^**^	0.90^**^	0.64^**^	0.85^**^
Pea grain yield in PS	0.28^**^	0.30^**^	0.27^**^	0.35^**^

NS, *, ** *, correlation not different from zero and different from zero at P < 0.05 and P < 0.01, respectively*.

Genotype × growing condition interaction for pea yield was observed in both test years (*P* < 0.01) and implied fairly low consistency of genotype yield responses across conditions, as indicated by genetic correlation values slightly above 0.40 in both test years (Table 3). Pea yield in PS exhibited similar broad-sense heritability as in MS, because the advantage of smaller experiment error was counterbalanced by the disadvantage of smaller genetic variation in PS relative to MS (Table 3). As a result, the predicted efficiency of indirect selection based on yield in PS relative to direct selection in MS was largely determined by genetic correlation values, achieving only 44% in both test years (Table 3).

**Table 3:** Genetic (CV_g_) and experiment error (CV_e_) coefficient of variation and broad-sense heritability on a line mean basis (*H*^2^) for pea direct selection for grain yield in mixed stand with cereals (MS) and pea indirect selection for yield in MS based on yield or a pea selection index in pure stand (PS), genetic correlation (*r*_*g*_) between direct and indirect selection criteria, and predicted efficiency (*E*_*R*_) of indirect selection criteria in PS relative to direct selection in MS, based on data of 144 pea inbred lines in each of two cropping years.

**Selection criterion**	**CV_g_ (%)**	**CV_e_ (%)**	***H*** **^2^ ± SE^a^**	***r_g_*** **± SE^b^**	***E_R_*** **(%)^c^**
***Year 2018–19***								
Pea grain yield in MS	33	44	0.62 ± 0.05		100
Pea grain yield in PS	24	27	0.70 ± 0.04	0.42 ± 0.11	44
Pea selection index in PS^d^	51	27	0.91 ± 0.01	0.72 ± 0.06	87
***Year 2019–20***								
Pea grain yield in MS	46	51	0.71 ± 0.04		100
Pea grain yield in PS	17	18	0.72 ± 0.04	0.43 ± 0.10	44
Pea selection index in PS^d^	15	13	0.80 ± 0.03	0.90 ± 0.05	96

a * Value ± standard error*.

b * Value ± standard error. Genotype × condition (MS or PS) interaction for pea yield significant at P < 0.01*.

c * E_R_ = [(H_PS_ / H_MS_) r_g_] × 100, where H_PS_ and H_MS_ are the square root of H^2^ for the relevant selection criteria in PS and MS, respectively*.

d * Selected from traits in PS associated with pea yield in MS; equal to: −1.413 + (0.0184 × pea plant height [in cm]) + (0.0962 × pea yield [in t/ha]) + (0.0476 × pea onset of flowering [in dd from April 1])*.

The large impact on pea yield responses of specific adaptation to MS or PS conditions was confirmed by estimates of variance components for grain yield across cropping years. While all genotypic and genotype × environment interaction components of variance were different from zero (*P* < 0.01), the variance of genotype × growing condition interaction was nearly two-fold larger than the genotypic variance, was definitely larger than the genotype × cropping year interaction variance, and was somewhat larger than the genotype × growing condition × year interaction variance (Supplementary Table 2). The occurrence of interaction of genotype with the year factor reduced the ability of performance data assessed in one year to predict genotype responses in an independent year.

The correlation of pea morphophysiological characteristics (as measured in PS) with pea yield was significantly different (*P* < 0.01) across MS and PS conditions for three traits, namely, onset of flowering, plant height at onset of flowering, and susceptibility to ascochyta blight (Table 4). In both test years, later onset of flowering and taller plant height were associated with pea yield in MS, while being poorly associated or not associated with pea yield in PS (Table 4). In contrast, greater susceptibility to ascochyta blight was strongly associated with lower pea yield in PS but not in MS (Table 4). Accordingly, relatively better yield response in MS as indicated by greater values of the pea MS/PS grain yield ratio was correlated with later onset of flowering and taller plant height (Table 4). The positive correlation of the MS/PS yield ratio with susceptibility to ascochyta blight (Table 4) indicated that relatively better performance in PS was associated with greater tolerance to the disease. Taller plant at onset of flowering (expected to be a key trait to compete for light), later flowering onset (contributing to maturity matching with associated cereals), and greater yield in PS were selected in this order as components of a selection index for greater pea yield in MS based on traits in PS. These traits were all significant at *P* < 0.001 in a stepwise multiple regression as a function of genotype yield in MS and jointly explained nearly 60% of the genotype variation. Their correlation to each other (*r* < |0.78|) was safely below any risk of collinearity. The selection index equation for pea yield in MS based on traits recorded in PS was:

**Table 4:** Phenotypic correlation of pea grain yield in mixed stand with cereals (MS) or in pure stand (PS) and ratio between MS and PS for pea grain yield with pea morphophysiological traits in PS, for 144 pea inbred lines grown in two cropping years.

	**Pea grain yield**							
	**2018–19**			**2019–20**			**MS/PS pea yield ratio**	
**Trait**	**MS^a^**	**PS^a^**	***u*** **test^b^**	**MS^a^**	**PS^a^**	***u*** **test^b^**	**2018–19**	**2019–20**
Onset of flowering	0.46**	−0.13 NS	**	0.50 **	−0.03 NS	**	0.49 **	0.53 **
Plant height at onset of flowering	0.55**	0.12 NS	**	0.59**	0.31**	**	0.41**	0.50**
Individual seed weight	−0.08 NS	0.30**	**	−0.09 NS	0.18*	*	−0.29**	−0.16 NS
Maturity date	0.20*	0.37**	NS	−0.07 NS	−0.16 NS	NS	−0.14 NS	0.00 NS
Plant height at maturity	0.36**	0.41**	NS	0.44**	0.21*	*	−0.02 NS	0.38**
Winter plant survival	0.13 NS	0.27**	NS	–	–		−0.09 NS	–
Susceptibility to ascochyta blight	−0.22**	−0.57**	**	–	–		0.25**	–

a NS, *, **, correlation not different from zero and different from zero at P < 0.05 and P < 0.01, respectively.

b NS, *, **, correlation coefficients between growing conditions in the same cropping year not different and different at P < 0.05 and P < 0.01, respectively, according to u test.

−1.413 + (0.0184 × pea plant height [in cm]) + (0.0962 × pea yield [in t/ha]) + (0.0476 × pea onset of flowering [in dd from April 1]).

The mean pea response for three morphophysiological traits across PS and MS conditions in the only year when it was assessed indicated non-significant trends toward delayed onset of flowering and taller plant stature in MS relative to PS (Table 1). Genotype × growing condition interaction was significant (*P* < 0.05) for onset of flowering and seed weight, but the consistency of genotype responses across conditions was very high for all traits according to genetic correlation (*r*_*g*_ ≥ 0.93).

Genotype value according to the selection index assessed in PS exhibited high genetic correlation with genotype yield in MS (*r* ≥ 0.72; Table 3) and somewhat higher broad-sense heritability than yield in MS (particularly in the first year, when the favorable growing conditions emphasized the genotype variation for most component traits of the index and, thereby, the genetic variation for index value: Table 3). As a result, the predicted efficiency of index-based selection in PS was in the range 87–96% relative to direct selection in MS (Table 3).

The comparison of direct vs. indirect selection strategies for predicted efficiency reported in Table 3 was relative to independent assessments for each test year and, as such, could not take account of possible differences among selection criteria for extent of genotype × location or genotype × year interactions (which ought to be minimal for an ideal selection criterion). Indeed, the selection index exhibited the additional advantage of lower genotype × year interaction (as shown by greater genetic correlation across years for genotype values) relative to both yield-based criteria (Table 5). This feature and its low experiment error (Table 3) contributed to higher broad-sense heritability over years of this criterion relative to yield-based criteria (Table 5). The comparison of selection strategies for predicted efficiency based on the size of phenotypic correlations between genotype value for the relevant selection criterion in a selection year and genotype yield in MS in another year could account for the advantage represented by lower genotype × year interaction for the selection index. This comparison revealed an average predicted efficiency advantage of 19% for this criterion relative to direct selection based on pea yield in MS (Table 6). The advantage of this criterion was greater for the selection year 2018–19 than for 2019–20 (Table 6), in coincidence with the much greater genetic variation that emerged for the selection index in the former year relative to the latter (Table 3). The predicted efficiency of yield-based selection in MS was about two-fold that of yield-based selection in PS according to this comparison (Table 6).

**Table 5:** Genetic correlation (*r*_*g*_) across two cropping years and broad-sense heritability over years on a line mean basis (*H*^2^) of pea yield in mixed stand with cereals (MS) and pea yield or a pea selection index in pure stand (PS), for 144 pea inbred lines across two cropping years.

**Trait^a^**	***r_g_*** **± SE**	***H*** **^2^**
Pea grain yield in MS	0.72 ± 0.09	0.619
Pea grain yield in PS	0.74 ± 0.09	0.581
Pea selection index in PS^b^	0.93 ± 0.03	0.696

a * Genotype × year interaction always significant at P < 0.01*.

b * See footnote d in Table 3 for index definition*.

**Table 6:** Phenotypic correlation (*r*) of pea genotype value in one year (selection year) with pea grain yield in mixed stand with cereals (MS) in another year (target environment) for three selection criteria based on MS or pure stand (PS) selection for individual selection years and averaged across selection years, and average predicted efficiency (*E*_*R*_) of selection criteria in PS relative to selection in MS, based on data of 144 pea inbred lines over two cropping years.

**Selection criterion**	***r*** **value**			***E_R_*** **(%)^a^**
	**Selection in 2018–19**	**Selection in 2019–20**	**Average**	
Pea grain yield in MS	0.475	0.475	0.475	100
Pea grain yield in PS	0.175	0.321	0.248	52
Pea selection index in PS^b^	0.663	0.472	0.568	119

a * E_R_ = (r_PS_ / r_MS_) ×100, where r_PS_ and r_MS_ are average r values for relevant PS and MS selection criteria, respectively*.

b * See footnote d in Table 3 for index definition*.

The alternative comparison of selection strategies based on actual yield gains performed by using by turns one year for selection and the other year for evaluation of yield gains also took account of genotype × year interaction effects. This comparison of selection criteria differed from that reported in Table 6 not only because it was based on actual yield gains but also because the selection was performed within each individual RIL population (reporting results averaged across populations: Table 7) rather than across the entire set of lines. Its results, averaged across selection years, indicated the similar efficiency of the index-based selection criterion in PS and the direct selection for pea yield in MS, as well as 64% efficiency of yield-based selection in PS relative to yield-based selection in MS (Table 7). Also here, the selection index-based criterion exhibited greater efficiency when selecting in the first year than in the second (Table 7).

**Table 7:** Mean grain yield and actual yield gain in mixed stand with cereals (MS) of pea lines selected within each of six recombinant inbred line (RIL) populations according to three phenotypic selection (PhS) criteria based on MS or pure stand (PS) selection and one genomic selection (GeS) criterion by performing PhS or GeS model training in one year and assessing yield gains of selected material in another year, and efficiency (*E*_*R*_) of selection criteria relative to PhS selection in MS, based on data of 144 pea inbred lines grown in two cropping years.

	**Selection/model training in 2018–19;** **yield assessment in 2019–20^a^**			**Selection/model training in 2019–20;** **yield assessment in 2018–19^a^**			
**Selection criterion**	**Mean yield (t/ha)**	**Yield gain (t/ha)**	***E_R_*** **(%)^b^**	**Mean yield (t/ha)**	**Yield gain (t/ha)**	***E_R_*** **(%)^b^**	***AverageE_R_*** **(%)**
PhS for pea grain yield in MS	1.310	0.476	100	1.205	0.397	100	100
PhS for pea grain yield in PS	1.105	0.271	57	1.090	0.282	71	64
PhS by a selection index in PS^c^	1.419	0.585	123	1.097	0.289	73	98
GeS for pea grain yield in MS^d^	1.282	0.448	94	1.129	0.321	81	88

a * Selection of two lines out of 23 for each RIL population; yield gain of the 12 selected lines over the mean value of six parent lines of the RIL populations*.

b * E_R_ = (G_SC_ / G_MS_) ×100, where G_SC_ and G_MS_ are yield gains for relevant selection criterion and for PhS for pea yield in MS, respectively*.

c * See footnote d in Table 3 for index definition*.

d * Selection within each population based on Ridge regression BLUP model training on 80% of the lines and estimation of breeding values for selection on the remaining 20% of the lines, rotating the folds and repeating the process 100 times to obtain stable predictions of top-yielding lines*.

### Genome-Enabled Predictions, Comparison of Genomic vs. Phenotypic Selection Strategies, and Genome-Wide Association Study

Next generation sequencing produced, on average, 2.2 M reads per genotype sample. The selected model configuration issued by the first step of genomic model tuning retained the thresholds of 0.05 for SNP missing data per marker and 0.50 for SNP missing data per genotype. This configuration, which was associated with 5,909 polymorphic SNP markers, was selected among those implying no loss of genotype samples because it maximized the average intra-RIL population inter-environment predictive ability for pea yield in MS (albeit with negligible difference to two configurations with lower SNP missing data per marker) while providing a reasonably high number of markers for the GWAS. More stringent thresholds of SNP missing data per genotype led to exclusion of some genotype samples without producing a substantial increase of intra-population predictive ability, as indicated by results in Supplementary Figure 1. This figure also showed the presence of variation among RIL populations for intra-population predictive ability. The selected configuration was adopted for the step of model tuning aimed to selection of the statistical model. Four models, i.e., Ridge Regression BLUP, Bayes A, Bayes Cπ and Reproducing kernel Hilbert space, displayed average intra-population predictive ability for pea yield in MS around 0.26, whereas Bayesian Lasso displayed slightly lower predictive ability (0.25). We selected the first model in view of its greater computational speed.

The predictive ability for intra-population inter-environment prediction of pea yield in MS averaged 0.267, while ranging from 0.183 (for progeny lines of Alliance × Isard) to 0.385 (for progeny lines of Kaspa × Isard) (Supplementary Table 3). No distinct relationship emerged between intra-population predictive ability and number of polymorphic markers or within-population phenotypic variation, although the RIL population with the highest predictive ability also displayed the highest number of polymorphic markers (Supplementary Table 3). The average predictive ability of populations was reduced by 27% (0.195 vs. 0.267) for the challenging scenario of inter-population inter-environment prediction (Table 8). In contrast, high predictive ability (0.532) was achieved for inter-environment predictions regarding the entire set of lines (considered as a unique genetic base) (Table 8). In all cases, model training on the data of the first year provided better predictions than training on the data of the second year (Table 8).

**Table 8:** Predictive ability of genomic selection models for pea grain yield in mixed stand with cereals using one cropping year for model training and another year for model validation, for (a) intra-population predictions for each of six individual recombinant inbred line (RIL) populations, (b) inter-population predictions for individual RIL populations, and (c) predictions for all genotypes neglecting population strata.

	**Predictive ability^a^**		
**Prediction**	**Training year 2018–19**	**Training year 2019–20**	**Average**
Intra-population^b^	0.326	0.208	0.267
Inter-population^c^	0.275	0.115	0.195
All genotypes^d^	0.625	0.438	0.532

a * As correlation of predicted values according to the Ridge regression BLUP with observed values, using data of 144 pea inbred lines*.

b * Averaged across 100 repetitions of five-fold stratified cross validations applied to each population; results for individual years averaged across populations*.

c * Model training on all data of the non-target populations; results for individual years averaged across populations*.

d * Averaged across 100 repetitions of five-fold stratified cross validations applied to all lines*.

A comparison of genomic vs. phenotypic selection strategies was performed for the two main contexts envisaged by earlier comparisons of phenotypic strategies. One was relative to selection among all genotypes, with predicted efficiency estimated from the size of the correlation between genotype values for the relevant selection criterion in one selection year and genotype yields in MS in an independent year. Relevant correlation values for this scenario are given in Table 6 for phenotypic selection criteria, and by correlations between cross validation-based genotype values issued by model training in one year and genotype yields in MS in an independent year as expressed by predictive ability values for all genotypes in Table 8 for genomic selection. The comparison based on correlation values averaged across years revealed 12% greater predicted efficiency of genomic selection relative to direct phenotypic selection for pea yield in MS (0.532 vs. 0.475), and 6% lower predicted efficiency of genomic selection relative to phenotypic index-based selection in PS (0.532 vs. 0.568) (Tables 6, 8). The second context for comparison of genomic vs. phenotypic selection strategies was relative to selection within each RIL population, with relative efficiency estimated according to actual yield gains. Results averaged across test years indicated 10−12% lower efficiency of genomic selection relative to best-performing phenotypic selection strategies as represented by selection for yield in MS and index-based selection in PS (Table 7).

**Figure 1:**
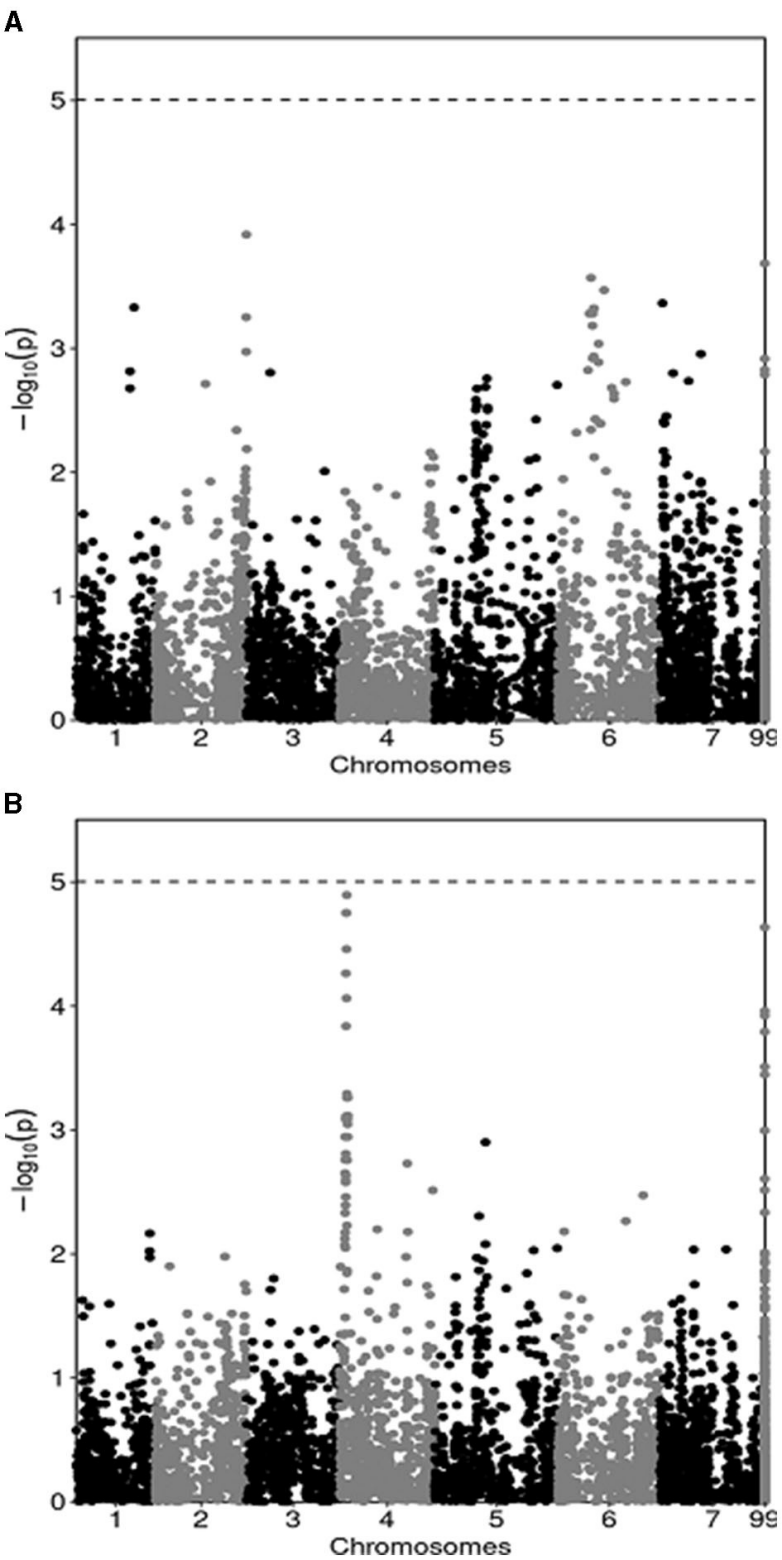
Manhattan plots showing the association score of SNP markers along pea chromosomes with pea grain yield in mixed stand with cereals **(A)** and in pure stand **(B)** in a genome-wide association study based on yield data of 144 lines averaged across two cropping years. The dashed line represents the Bonferroni threshold at *P* < 0.05.

The results of the GWAS are summarized by Manhattan plots reporting marker-trait associations relative to pea yield in MS (Figure 1A) and in PS (Figure 1B). They indicated many regions of the genome that featured a slight association, with no marker reaching the Bonferroni threshold for significant (*P* < 0.05) association. In agreement with the modest genetic correlation for pea genotype yield across MS and PS conditions, the GWAS revealed modest consistency across growing conditions for markers that tended to display some association with the yield trait. In particular, one genomic area on chromosome 4 whose association with yield in PS approached *P* < 0.05 significance (Figure 1B) showed no trend toward association with yield in MS (Figure 1A). Likewise, five genomic regions that tended toward association with yield in MS on the ground of association scores ≥ 3 (one each on chromosomes 1, 2 and 6, and two on chromosome 7: Figure 1A) showed no local peak for yield in PS (Figure 1B). Only one region on chromosome 5 tended toward association with yield in both growing conditions, albeit with a modest linkage (association score slightly below 3) (Figure 1).

## Discussion

Our preliminary study highlighted the difficulty to identify cereal companions with sufficient earliness of maturity for pea-cereal intercrops aimed to grain production. This result restricted the choice of cereal companion species and cultivars, and influenced the definition of pea traits contributing to specific adaptation to MS by promoting the advantage of a late pea phenology. The extent of pea-cereal mismatch of maturity may depend on the specific germplasm, cropping region and sowing season. For example, pea displayed a trend toward later maturity than barley (the earliest small-grain cereal) for locally well-adapted cultivars evaluated in Switzerland under spring sowing (B. Haug, personal communication, 2021). The phenological type of the selected pea parents that originated our genetic base included spring-type (e.g., Attika), Mediterranean (e.g., Kaspa) and winter-type (e.g., Isard) material. In autumn-sown Italian environments these cultivars exhibited moderate variation for onset of flowering along with modest variation for maturity time (Annicchiarico, 2005; Annicchiarico and Iannucci, 2008) due to the combined effect of terminal drought and high temperatures. The same response was displayed by their derived lines in the current study. Later pea phenology may be searched for by growing photoperiod-sensitive germplasm selected for central Europe to enhance pea winter hardiness (Lejeune-Hénaut et al., 2008), but this material is unlikely to be adapted to the warm and dry summers of southern Europe. Therefore, the identification and/or selection of early-maturing barley and wheat companions probably is the main avenue to obtain cereal companions compatible with pea for autumn-sown intercrops in our target region.

Harper's (1977) general observation that the yield efficiency of a mixture depends mainly on the performance of its weaker partner, which was confirmed by various experimental studies (Ofori and Stern, 1987), highlighted the importance of selecting for greater competitive ability the component species that is expected to be outcompeted under ordinary cropping conditions in a target region. From a plant breeding perspective, this conclusion is supported by the fact that the genetic correlation for genotype yield responses across MS and PS conditions tends to be lower in the presence of larger competitive stress exerted on the focus species (Annicchiarico and Piano, 1994). This study confirmed the severe competitive disadvantage reported for pea by earlier studies encompassing different cereal companions, target regions and sowing times (Jensen, 1996; Corre-Hellou et al., 2006; Lithourgidis et al., 2011; Annicchiarico et al., 2017). The value slightly above 0.40 of the genetic correlation for pea yield across MS and PS conditions was consistent across test years despite their differences for sowing time, N fertilization and cereal companions. This value was lower than the average value across studies on perennial legume-grass or annual legume-cereal intercrops in a recent review (Annicchiarico et al., 2019a). Likewise, the current predicted efficiency of indirect selection in PS relative to direct selection in MS based on results of single experiments was lower than the average one in early studies on legume-based intercrops, namely, 44% (Table 3) vs. 60% (Annicchiarico et al., 2019a). The observed increase of the genetic coefficient of variation for yield in MS relative to PS agrees with earlier results for grain (Atuahene-Amankwa and Michaels, 1997) and perennial legumes (Annicchiarico, 2003). The lack of substantially greater broad-sense heritability of MS relative to PS caused by a concurrent trend of MS toward greater experiment error than PS agrees as well with earlier findings for legume-based intercrops (Annicchiarico et al., 2019a).

The GWAS provided an unprecedented genome-based insight and justification for the modest genetic correlation for genotype yields across MS and PS conditions that emerged in a quantitative genetics framework. The presence of many genomic regions displaying a slight, non-significant association was expected for a complex, highly polygenic trait such as grain yield. The large inconsistency across growing conditions for markers that tended to display some association with the yield trait emerged clearly from the overview of association scores in Manhattan plots. In this study the GWAS did not aim to discover quantitative trait loci, given the limited practical interest of marker-assisted selection compared with genomic selection for the improvement of largely polygenic traits (Bernardo and Yu, 2007).

The only modest decrease of competitive stress exerted on pea in the second year relative to the first year suggested that pea competitive disadvantage is ordinary in the target region and is not easy to be overcome just by agronomic decisions relative to sowing time, N fertilization or cereal companion. Other considerations support the greater perspective interest of pea breeding over crop management to improve pea-cereal intercrops. While more balanced grain legume-cereal mixtures could be obtained by adopting less vigorous cereal companions, no N fertilization or increased legume sowing rate (Ofori and Stern, 1987; Yu et al., 2016), these technical choices may produce lower total yield of the mixture compared with the adoption of a legume component with increased competitive ability. This conclusion is supported by: (a) several reports highlighting the importance of N fertilization for the agronomic and economic performance of grain legume-cereal mixtures (e.g., Hauggaard-Nielsen and Jensen, 2001; Kiwia et al., 2019); (b) results for perennial crops indicating that total mixture yield tends to be maximized by pairs of components characterized by the highest and most similar plant vigor (Zannone et al., 1986; Annicchiarico and Piano, 1994). The latter results agree with the current finding that pea lines with greater competitive ability tend to produce mixtures not only more balanced but also better yielding.

The large inconsistency across MS and PS conditions of correlations of pea morphophysiological traits with grain yield shed light on useful pea adaptive traits for intercropping. Taller plant stature at onset of flowering was the main trait in this respect according to correlation results and the selection of this trait as the first one in the stepwise regression analysis leading to definition of the selection index. Taller plant is generally associated with greater competitive ability of erect plants under moderately favorable growing conditions (Keddy, 1990), owing to its crucial importance in competition for light. Taller pea plants exhibited greater competitive ability in different pea-grain legume associations assessed by simulation (Barillot et al., 2012, 2014) and field studies (Hauggaard-Nielsen and Jensen, 2001; Annicchiarico et al., 2012, 2017). The usefulness of later onset of flowering for adaptation to MS descends from the discussed trend toward mismatched maturity of pea and associated cereals and the much narrower variation of a more relevant trait in this context such as pea maturity date. As anticipated, the relationship of phenology with yield response in MS is expected to be germplasm- and environment-specific. The selection of pea yield in PS as a third trait in the index of selection for pea yield in MS agrees with the positive genetic correlation for yield across the two conditions, which implies that a portion of the variation for intrinsic pea grain yielding ability (as indicated by PS performance) is also relevant to MS performance. The greater importance of tolerance to ascochyta blight in PS than in MS agrees with the fact that tolerance to pests and diseases is usually less important in MS, because of the dilution of host density allowed for by the associated species (Boudreau, 2013).

The assessment only in one year of morphophysiological traits across MS and PS conditions provided only preliminary indications on pea phenotypic plasticity in response to intercropping. Recalling that phenotypic plasticity is the ability of a genotype to alter its trait values in response to environmental conditions (Bradshaw, 1965), pea displayed only limited and non-significant shifts of trait mean value passing from PS to MS (albeit in the adaptively meaningful directions of delayed onset of flowering and taller plant stature). While these results concerned the mean response of pea, phenotypic plasticity responses of practical interest for breeders relate to genetic variation as revealed by genotype × growing condition interaction for a focus trait that is associated with relatively better performance in MS. For example, interaction effects relative to white clover genotypes with greater capacity of petiole elongation in MS were indicative of better phenotypic plasticity-based adaptation to intercropping with vigorous grasses (Annicchiarico, 2003), as a consequence of a phytochrome-mediated mechanism for shade avoidance that is present in white clover (Robin et al., 1992) and may affect various vegetative organs in other species (Schmitt et al., 2003). In this study, the highly consistent genotype responses across PS and MS conditions suggest quite limited variation for phenotypic plasticity of pea plant height at flowering onset or other observed traits.

The observed small difference in predictive ability among various statistical models usable for genomic selection was reported earlier for pea yield (Annicchiarico et al., 2019b) or other pea traits (Burstin et al., 2015). The good performance of Ridge regression BLUP agrees with its suitability for traits influenced by a large number of minor genes, such as grain yield (Wang et al., 2018).

The training set for genomic selection was the same for intra-population selection and for all-genotype selection (i.e., selection within the whole set of genotypes without distinction between RIL populations), always including the parent lines and 80% of the inbred lines per RIL population. The two-fold greater genome-enabled predictive ability for the latter selection scenario relative to the former (0.532 vs. 0.267: Table 8) descended from the possibility to also exploit the phenotypic variation due to mean differences between populations and the wider molecular variation provided by the pooled populations. The average intra-population inter-environment predictive ability for pea yield in MS was only somewhat lower than that observed for pea yield in PS across Italian environments for a subset of three of the current RIL populations, which was equal to 0.296 (Annicchiarico et al., 2019b).

The adoption of different conditions for MS testing in the two test years probably inflated the extent of genotype × growing condition × year interaction. However, such diverse conditions reflected better the diversity of possible intercropping conditions in the target region, thereby providing a more realistic (albeit more challenging) scenario for the comparison of phenotypic or genomic selection strategies based on selection in one year and assessment of yield gains in an independent year. We envisaged two main selection scenarios for these comparisons, namely (a) all-genotype selection (with comparison based on predicted yield gains), and (b) selection within each RIL population (with comparison based on actual yield gains from selection of two lines out of 23 per population). Considering direct phenotypic selection in MS as the benchmark for comparison of alternative selection strategies, our results suggested that (a) the relative efficiency of 52–64% exhibited by indirect selection based on pea yield in PS is too low to be compensated by budget savings arising from no need for pea proportion assessment; (b) the index-based selection in PS provided a valuable alternative to selection for yield in MS, particularly when selecting across RIL populations (where it displayed 19% greater predicted efficiency), when considering that the additional morphophysiological traits to be recorded beside yield, i.e., onset of flowering and plant height at flowering onset, are less expensive to record than the assessment of pea proportion in MS; (c) genomic selection for pea yield in MS has high interest for selection across or within RIL populations, because its efficiency was comparable to phenotypic selection for yield in MS and would definitely be greater when taking into account the effect on predicted or actual gains per unit time of its shorter selection cycle and smaller evaluation cost per genotype. In particular, the ability by genomic selection to perform two selection cycles per year would imply efficiency values relative to selection for yield in MS of 176% and 224% based on actual and predicted gains, respectively, per unit time. The double amount of evaluated genotypes per year assumed for genomic selection would be supported by at least two-fold lower evaluation cost per genotype compared with phenotypic selection in MS, according to a GBS fee of about € 60 (including taxes) and an estimated cost for one-year phenotypic selection in MS of about € 120–130. We did not formally assess the relative merit of genomic selection based on inter-population inter-environment predictions, but the 27% average loss of predictive accuracy suggests that even this selection strategy may be efficient for pea selection aimed to intercropping.

In conclusion, this study highlighted the importance of pea selection for intercropping as a means to obtain more balanced and more productive pea-cereal intercrops, and indicated the high efficiency in this context of phenotypic selection for pea yield in MS, genomic selection for the same trait, and indirect phenotypic selection based on a selection index of traits related to pea competitive ability that are assessed in PS. While many studies investigated the relationship of competitive ability with morphophysiological traits in legume species, just a few provided a formal assessment of the efficiency of trait-based indirect selection relative to yield-based selection (Annicchiarico et al., 2019a). In addition, our study provided unprecedented evidence for the value of genomic selection for intercropping on the basis of experimental data. Interestingly, the about two-fold greater efficiency of genomic selection relative to phenotypic selection for yield in MS according to yield gains per unit time is close to the 2.3-fold advantage predicted for genomic selection by Bančič et al. (2021) according to simulation results for the current scenario of genetic correlation around 0.4 across MS and PS conditions. Genomic selection may display the highest efficiency but requires an initial stage of germplasm evaluation in MS for model training which can, anyway, be used for phenotypic selection purposes. A possible limitation of our findings was the limited sampling of test environments that our estimates of predicted or actual yield gains were based upon. More conclusive indications are expected from future research work aimed to compare the current selection strategies in terms of actual yield gains in MS over a larger number of test environments.

## Data Availability Statement

The original contributions generated for the study are publicly available. This data can be found here: National Center for Biotechnology Information (NCBI) BioProject database under accession number PRJNA744476.

## Author Contributions

PA designed the research work and obtained financial resources. LP and PA were responsible for, and TN, MR, and CM contributed to, phenotyping experiments. BF was responsible for the extraction and preparation of DNA for outsourced analyses. PA was responsible, and CM contributed to, phenotypic data analyses. NN was responsible for molecular data analysis. PA drafted the manuscript. All authors revised and approved the manuscript.

## Funding

This work was carried out within the Horizon 2020 project “Redesigning European cropping systems based on species mixtures—REMIX” funded by the European Union (Grant Agreement no. 727217). Genotyping data were provided by the PRIMA project “Research-based participatory approaches for adopting conservation agriculture in the Mediterranean area—CAMA” supported by the European Union (Grant Agreement no. 1912).

## Conflict of Interest

The authors declare that the research was conducted in the absence of any commercial or financial relationships that could be construed as a potential conflict of interest.

## Publisher's Note

All claims expressed in this article are solely those of the authors and do not necessarily represent those of their affiliated organizations, or those of the publisher, the editors and the reviewers. Any product that may be evaluated in this article, or claim that may be made by its manufacturer, is not guaranteed or endorsed by the publisher.
