# Supplementary material for: Pea Breeding for Intercropping With Cereals: Variation for Competitive Ability and Associated Traits, and Assessment of Phenotypic and Genomic Selection Strategies
Source: Front Plant Sci. 2021 Sep 23;12:731949. doi: 10.3389/fpls.2021.731949 (PMC8495324; doi:10.3389/fpls.2021.731949)
Supplement: Supplementary file 1 [file Table_1.DOCX]

**Supplementary Table 1. Variation for heading (cereals) or onset of flowering (pea) date, maturity date and plant height at heading/onset of flowering of 20 varieties or breeding lines of barley, bread wheat, durum wheat and triticale and 14 varieties or breeding lines of pea**

| Species | Variety / line | Origin | Heading/onset of flowering (dd from April 1) | Maturity (dd from April 1) | Plant height (cm) |
| --- | --- | --- | --- | --- | --- |
| *Cereal genotypes* |  |  |  |  |  |
| Barley | Alimini | Italy | 29 | 75 | 90 |
| Barley | Aragona | France | 28 | 75 | 89 |
| Barley | Atlante | Italy | 24 | 69 | 92 |
| Bread wheat | A208 | France | 32 | 80 | 102 |
| Bread wheat | A210 | France | 33 | 81 | 117 |
| Bread wheat | Almeria | Italy | 32 | 81 | 73 |
| Bread wheat | Antonello | Italy | 29 | 78 | 84 |
| Bread wheat | Apache | France | 36 | 82 | 73 |
| Bread wheat | F426 | France | 30 | 80 | 103 |
| Bread wheat | Fanion | France | 34 | 82 | 68 |
| Bread wheat | Goucourt | France | 36 | 82 | 65 |
| Bread wheat | Insegrain | France | 36 | 82 | 73 |
| Bread wheat | Salviter | Italy | 29 | 80 | 77 |
| Bread wheat | San Pastore | Italy | 29 | 72 | 102 |
| Bread wheat | Solehio | Italy | 32 | 79 | 74 |
| Bread wheat | Spada | Italy | 26 | 72 | 69 |
| Bread wheat | Tulip | France | 36 | 82 | 81 |
| Durum wheat | Ciclope | Italy | 33 | 88 | 71 |
| Triticale | Flash | France | 23 | 80 | 95 |
| Triticale | Vivacio | France | 25 | 81 | 110 |
| LSD (*P* < 0.05) |  |  | 2 | 2 | 7 |
| Mean value^a^ |  |  | 30.6 | 79.0 | 85.4 |
| Range values |  |  | 23-36 | 69-88 | 65-117 |
| *Pea genotypes* |  |  |  |  |  |
| Mean value^a^ |  |  | 26.7 | 64.9 | 68.7 |
| *Pea variety subset*^b^ |  |  |  |  |  |
| Mean value |  |  | 25.3 | 64.7 | 66 |
| Range values |  |  | 24-29 | 62-67 | 50-77 |
| *Pea breeding line subset* |  |  |  |  |  |
| Mean value |  |  | 27.7 | 65.0 | 71 |
| Range values |  |  | 22-32 | 62-67 | 50-81 |

^a^ Cereal mean value different from pea mean value at *P* < 0.01 for all traits.

^b^ Alliance, Attika, Dove, Guifilo, Isard and Kaspa.
