# Supplementary material for: Pea Breeding for Intercropping With Cereals: Variation for Competitive Ability and Associated Traits, and Assessment of Phenotypic and Genomic Selection Strategies
Source: Front Plant Sci. 2021 Sep 23;12:731949. doi: 10.3389/fpls.2021.731949 (PMC8495324; doi:10.3389/fpls.2021.731949)
Supplement: Supplementary file 2 [file Table_2.DOCX]

**Supplementary Table 2. Analysis of variance and estimation of variance components for grain yield of 144 pea inbred lines grown in the conditions of pure stand and mixed stand with cereals for two cropping years**

| Source of variation | Degrees of freedom | Mean square | *F* test^a^ | Variance component |
| --- | --- | --- | --- | --- |
| Year (Y) | 1 | 232.45 | * | – |
| Block (B) in Y | 4 | 20.16 | – | – |
| Condition (C) | 1 | 7014.51 | ** | – |
| C x Y | 1 | 271.27 | ** | – |
| C x B (Y) | 4 | 11.00 | – | – |
| Genotype (G) | 143 | 5.42 | ** | 0.16 |
| G x C | 143 | 3.60 | ** | 0.34 |
| G x Y | 143 | 1.94 | ** | 0.04 |
| G x C x Y | 143 | 1.77 | ** | 0.27 |
| Pooled error | 1611 | 1.03 | – | 1.03 |

^a^ *, ** = significant at *P* < 0.05 and *P* < 0.01, respectively.
