# Supplementary material for: Pea Breeding for Intercropping With Cereals: Variation for Competitive Ability and Associated Traits, and Assessment of Phenotypic and Genomic Selection Strategies
Source: Front Plant Sci. 2021 Sep 23;12:731949. doi: 10.3389/fpls.2021.731949 (PMC8495324; doi:10.3389/fpls.2021.731949)
Supplement: Supplementary file 3 [file Table_3.DOCX]

**Supplementary Table 3. Values averaged across two test years of intra-population inter-environment predictive ability for pea grain yield in mixed stand and coefficient of variation of BLUP phenotyping data used for genomic selection, and number of polymorphic markers, for six recombinant inbred line populations**

| RIL population parents | Predictive ability^a^ | Coefficient of variation | Number of markers |
| --- | --- | --- | --- |
| Attika × Guifilo | 0.303 | 0.254 | 2,429 |
| Attika × Isard | 0.212 | 0.284 | 3,877 |
| Alliance × Isard | 0.183 | 0.275 | 3,833 |
| Dove × Attika | 0.196 | 0.223 | 3,439 |
| Kaspa × Attika | 0.325 | 0.225 | 4,177 |
| Kaspa × Isard | 0.385 | 0.252 | 4,712 |

^a^ According to the Ridge Regression BLUP model using data of 144 pea inbred lines; values averaged across 50 repetitions of five-fold stratified cross validations applied to each population.
