# Supplementary figures and images for: Pea Breeding for Intercropping With Cereals: Variation for Competitive Ability and Associated Traits, and Assessment of Phenotypic and Genomic Selection Strategies
Source: Front Plant Sci. 2021 Sep 23;12:731949. doi: 10.3389/fpls.2021.731949 (PMC8495324; doi:10.3389/fpls.2021.731949)

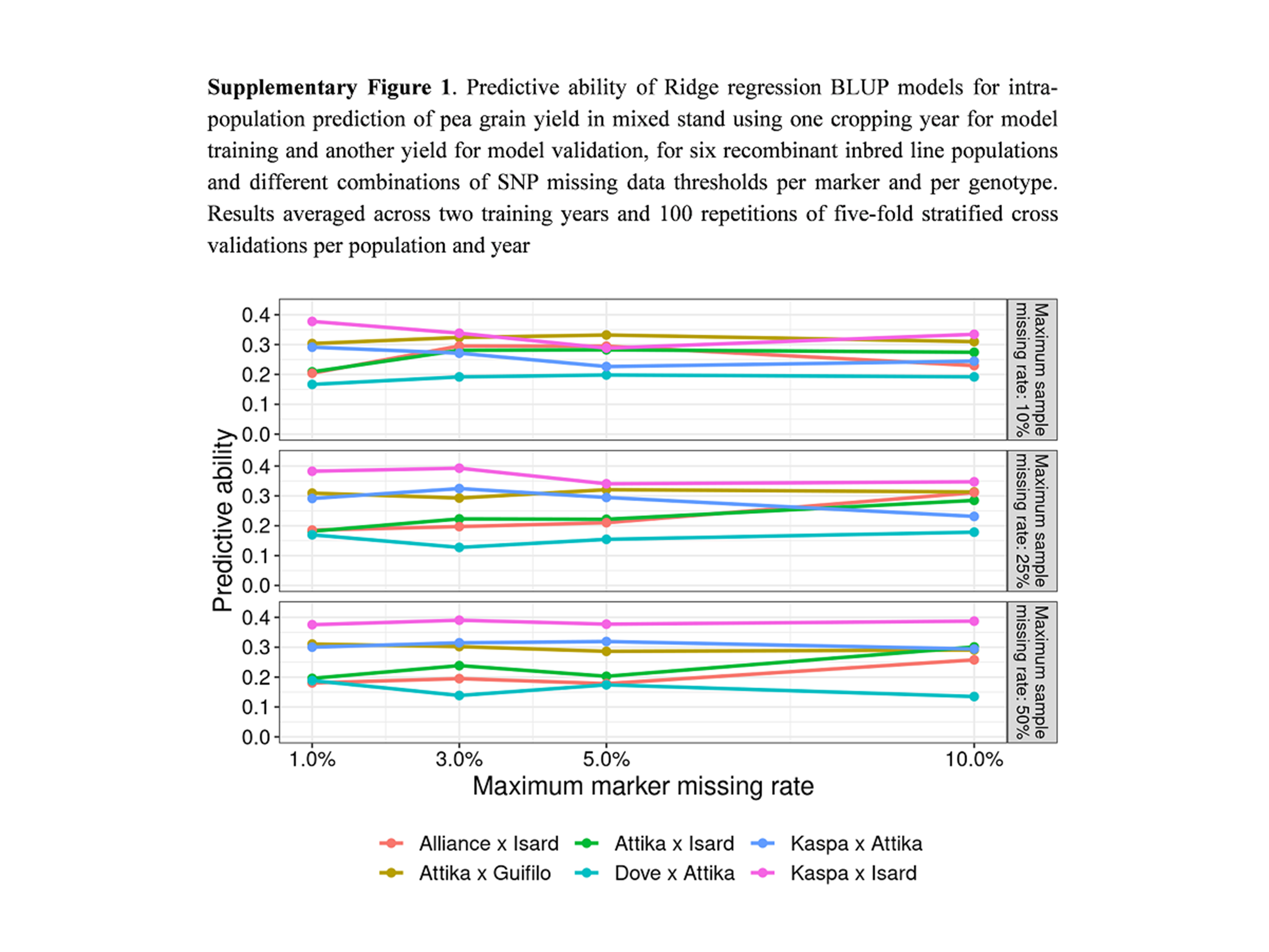

Supplement: Supplementary file 4 [file Image_1.TIF]
